# Online neuropilates classes in chronic stroke patients: Protocol for a randomised controlled feasibility study

**DOI:** 10.1016/j.conctc.2023.101068

**Published:** 2023-01-20

**Authors:** Eimear Cronin, Kenneth Monaghan

**Affiliations:** a Neuroplasticity Research Group, ATU Sligo, Ash Lane, Ballytivnan, Sligo, Ireland; b Physiotherapy Department, St. John's Hospital, Sligo, Ireland

**Keywords:** Stroke, Pilates, Protocol, Rehabilitation, Physiotherapy

## Abstract

**Introduction:**

Stroke survivors often demonstrate low physical activity levels and experience barriers to physical exercise including embarrassment, low self-efficacy and a shortage of tailored community exercise programmes. Access to physical activity programmes for stroke survivors could be improved by providing tailored, online programmes, although little is known about the safety and feasibility of online exercise classes for stroke survivors. One such programme of exercise which has received little attention in the literature is neuropilates. Neuropilates is the practice of a modified pilates programme in those with neurological conditions and is theorised to have beneficial effects on strength, balance and proprioception in stroke survivors. No previous study has been conducted to investigate online, remotely supervised neuropilates exercise classes in the stroke survivors.

**Method and Analysis:**

This single assessor blinded randomised controlled feasibility study will compare a 6-week online, remotely instructed neuropilates programme to a 6-week online, remotely instructed generalised exercise programme and a 6-week unsupervised generalised home exercise programme in chronic stroke patients. Twenty adults, at least 6 months post stroke, and finished their formal rehabilitation will be recruited to the study. Primary feasibility outcome measures will include patient tolerance of the programme, adherence rates, adverse events, recruitment and retention. Secondary clinical outcomes will include; balance, gait, tone and quality of life. Assessments will be completed at baseline, on programme completion and 3 months post completion by a Physiotherapist blinded to the group allocation.

**Ethics and dissemination:**

This study has received ethical approval from the Sligo University Hospital Ethics committee and ATU ethics board. Results will be published in peer-reviewed journals and presented at national and international conferences.

The trial has been registered on clinicaltrials.gov (Identifier: NCT04491279).

## 1 Introduction

Stroke is a leading cause of disability worldwide [1]. The age-standardised mortality rates of stroke have decreased sharply since 1990 [2], but the decrease in incidence rates has been less steep [3], placing increasing pressures on rehabilitation and post-rehabilitation services. Stroke survivors are at high risk of developing disabilities including motor deficits and balance deficits with the risk of falls and fractures in this population being 50% higher than age matched controls [3]. Community dwelling stroke survivors are also less active than their age matched counterparts [4,5] and less active than others with chronic health conditions [6]. In this latter study by Ashe et al., 2009 [6], participants in the stroke cluster had the highest levels of inactivity (27%) compared to participants in the degenerative neurological disorders cluster (17%) and those with vascular or heart conditions (24%).

Stroke survivors have themselves cited some reasons for their lower physical activity levels, including; embarrassment, low self-efficacy, a shortage of tailored community exercise programmes and a lack of skilled exercise professionals [7,8]. The benefits of physical exercise for stroke survivors on balance, mobility and fitness have been well documented [[9], [10], [11]]. There is, therefore, a need for more specialised exercise programmes staffed by trained professionals, which may help stroke survivors to lead less sedentary lives, prevent the sequalae of inactivity and decrease the risk of further stroke.

One such programme of exercise which has been considerably under researched is Pilates. Created by Joseph Pilates in the 1920s, Pilates is a programme of mind-body exercises focussing on strength, core stability, flexibility, muscle control, posture and breathing [12]. Neuropilates is the practice of modified pilates programmes in those with neurological conditions [13]. Neuropilates uses the core principles of traditional pilates with modifications based on patient need and with an underpinning appreciation of neurological rehabilitation principles such as neuroplasticity and motor learning. Neuropilates may, for example, use the more functional positions of sitting and standing for exercises, may increase repetitions in line with current evidence on repetitive task training post stroke [14] and may use visualisation cues and motor imagery often in line with best practice [15]. The Australian Physiotherapy and Pilates Institute (APPI®) theorises that neuropilates has beneficial effects on strength, postural control, alignment, stability, balance, proprioception, coordination and gait in those with deficits due to a neurological condition through retraining low threshold activity of local muscles and decreasing over-active global muscles [16].

In the development of this neuropilates intervention, and in accordance with the MRC framework for intervention development [17], the theories underpinning the intervention, outlined in the previous paragraph were initially tested through a review of published research. A systematic literature review and meta-analysis of 5 randomised controlled trials investigating pilates in a combined total of 122 post stroke patients [18] found moderate evidence for improvements in balance and limited evidence for improvements in quality of life, cardiopulmonary function and gait with pilates interventions. However, the mean methodological score on the PEDro scale for all five studies was only 4.8, or “fair” quality and control groups were either inactive or participating in conventional rehabilitation only. There is, therefore, no direct evidence that pilates is superior to any other form of exercise, only that is may be superior to no intervention or standard care. No adverse events were reported during the five studies investigated and so initial evidence supports the safety of pilates in this patient group. Previous literature reviews have found pilates exercises to be also safely and effectively applied in elderly patients [19], in patients with non-communicable diseases [20] and in patients with multiple sclerosis [21].

With the emergence of the Covid 19 pandemic, vital community services and support systems for post stroke patients came to an abrupt stop as health professionals worldwide adapted to public health guidelines [22,23]. In response to the pandemic, there has been a rapid shift to telehealth services, with supervised exercise delivered remotely becoming an increasingly employed option [22]. There is some preliminary data to support the safety and efficacy of virtual pulmonary rehabilitation and virtual cardiac rehabilitation programmes [24,25], but less is known about virtual exercise classes for stroke survivors. A recent Cochrane review of 22 trials investigating all forms of telerehabilitation in a total of 1937 stroke survivors found evidence to suggest that telerehabilitation may not be inferior to in-person therapy and appears to be “a reasonable model of service delivery” [26].

To date, no study has been conducted to investigate online, remotely supervised neuropilates exercise classes in the stroke survivors. In line with the MRC framework for development of complex interventions [17], stakeholders were involved in the development of our online neuropilates interventions. Surveys were conducted with Physiotherapists and patients who had either conducted or undergone a Physiotherapy-led online exercise class to explore their experiences, perspectives and ideas to improve future services. The results of these surveys are currently in press and demonstrate that satisfaction with online exercise classes was high amongst patients with 95% reporting they were moderately or extremely satisfied. Both Physiotherapists and patients stated that the main disadvantages of online exercise classes were a lack of interaction between participants and reduced direct observation by the Physiotherapists. Many patients worried that they were doing the exercises wrong but couldn't be observed fully in the online format. The results of this stakeholder engagement were taken into consideration when designing our intervention and based on feedback from these surveys, it was decided that assessments would take place face-to-face and include an initial education session on technological aspects of logging into the class as well as the formative pilates position of neutral posture with transversus abdominis activation [27].

The intervention was then further shaped by the results of a case study which was carried out [28]. Results from this case study demonstrated that online, remotely supervised neuropilates training is feasible and safe in a one-to-one scenario with a stroke survivor [28] but safety and efficacy have not yet been demonstrated with higher instructor to participant ratios. Also, pilates exercises have never been compared in the literature to general exercise in stroke survivors, which calls into question their superiority, if any, over other forms of exercise.

The proposed randomised controlled feasibility study will begin to address some of the afore mentioned gaps in the literature base and determine whether the intervention should be recommended for efficacy testing in line with the standards set for feasibility trials by Bowen et al., 2009 [29]. The feasibility study has been registered on clinicaltrials.gov (Identifier: NCT04491279). The aim of the study is to conduct a randomised controlled feasibility study to examine the feasibility and clinical outcomes on stroke survivors of a live, online, once weekly, 6-week neuropilates exercise class when compared to two control groups; the first control group will be participating in a live, online, once weekly, supervised, 6-week generalised exercise class, and the second will be given a home exercise programme to carry out unsupervised. The objectives of the study are as follows;

1. To examine the feasibility of conducting a 6-week online exercise classes in stroke survivors, including acceptability, implementation, practicality, adaptation, adverse events, adherence rates, recruitment, retention and patient tolerance.
2. To examine the effects of a 6-week online remotely supervised neuropilates exercise class on gait, balance, tone, function and quality of life in stroke survivors compared to a 6-week online remotely supervised general exercises class.
3. To examine the effects of a 6-week online remotely supervised neuropilates exercise class on gait, balance, tone, function and quality of life in stroke survivors compared to a 6-week non-supervised generalised home exercise programme.

**Fig. 1:**
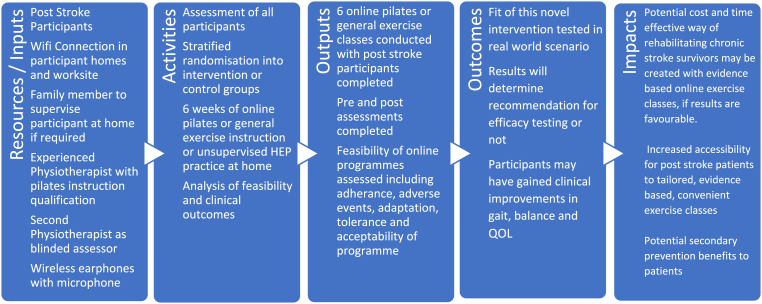
Logic model for intervention development.

We hypothesize that both online remotely supervised exercise groups may experience improvements in balance, mobility, strength, function and quality of life. We also hypothesize that the neuropilates group may have significantly greater improvements in balance and potentially gait outcomes than the control groups due to the results of a recent literature review [18]. This paper has been structured and written in accordance with the SPIRIT guidelines for clinical trial protocol papers [30]. A logic model for the proposed intervention can be seen in Fig. 1.

## 2 Research design and methods

### 2.1 Study design

The design of the trial is a single (assessor) blinded, randomised controlled feasibility study. Due to the nature of the intervention, neither the instructing therapist nor the participant can be blinded to group allocation. An independent assessor, who is blinded to the treatment assignment, will perform all clinical outcome assessments. Participants will undergo stratified random sampling, following an initial phone screening conversation. The sampling frame will be 20–25 potential participants. The age of the participants and functional status will be the strata considered during this randomisation process to ensure a representative sample in each group with factors that could influence outcome accounted for. In terms of functional ability, higher functionally independent participants will be considered to be those who can walk without an assistive device and lower functionally independent participants will be those who need an assistive device to walk. There will then be four strata from which random sampling will extract from; 1) Less than 65 years, lower functioning, 2) Greater than 65 years, lower functioning, 3) Less than 65 years, higher functioning, 4) Greater than 65 years, higher functioning. Participants from stratum 1 will be divided into intervention and control groups by the following method; all names will be placed into envelopes, shuffled and divided as evenly as possible into intervention and control groups by an independent researcher. This process will be repeated for the rest of the strata. This should result in an even, random spread from each stratum across all groups.

### 2.2 Study setting

Participants' will be exercising at home, with some connecting virtually to live, supervised classes. All clinical outcome assessments will take place face-to-face at the Physiotherapy Department at St. John's Hospital, Sligo, Ireland.

### 2.3 Participants/sample size

We will aim to recruit 20–25 adults with a diagnosis of stroke who meet the inclusion criteria outlined in Table 1. The patient must be diagnosed with stroke by a neurologist or gerontologist and confirmed by radiological imaging where applicable. The sample size chosen is based upon previous randomised control trials carried out in this field [[31], [32], [33]] as well as current pilot trial research recommendations [34]. The sample size chosen should be practical within this single centre trial to allow completion within a timely manner and should provide valuable preliminary information on practicalities of the intervention, feasibility, recruitment, uptake and attrition. The average attrition rate reported in stroke rehabilitation randomised controlled trials is 6% so it is likely our attrition rate will be similar [35].

**Table 1:** Selection criteria.

Inclusion Criteria	Exclusion criteria
1. Aged >18 years	1. Involvement in other studies or rehabilitation programmes.
2. Diagnosis of chronic stroke (>6 months post stroke event) and formal rehabilitation completed.	2. Severe cognitive deficits or difficulty following instructions.
3. Able to transfer and mobilise >10 m independently with or without assistive device	3. Significant hearing difficulties.
4. Internet access in their homes: Wifi or access to someone with mobile data	4. Significant visual deficit
5. Cognitive ability to understand the programme.	5. Uncontrolled pain or uncontrolled high blood pressure.

Participants will be recruited from outpatient physiotherapy clinics throughout Sligo, occupational therapy departments and also from geriatrician clinics. Community Physiotherapists, Occupational therapists and Geriatricians will act as gate keepers, identifying potential participants. They will provide potential participants with information sheets regarding the trial and ask potential participants to sign a consent to contact form. Once participant consent has been gained, participant information can then be passed on to the principal researcher. The principal researcher will then phone the participants to provide further information regarding the trial, carry out initial screening and provide a date for an initial face to face assessment. Informed consent will be sought from participants by the principal researcher at the initial assessment.

### 2.4 Selection criteria

Inclusion and exclusion criteria can be seen in Table 1.

### 2.5 Outcome measurement

Participants will be assessed pre-trial, post-trial and 3 months post trial. Primary feasibility outcomes will include recruitment, rates of attrition, adherence rates, adverse events, practicalities of the intervention and tolerance of the intervention.

An independent assessor, a CORU registered Physiotherapist with experience assessing stroke survivors, will carry out the secondary clinical standardised outcome measurements. Balance and gait will be assessed using the Tinetti Balance and Gait Assessment [36], the Timed Up and Go Assessment [37] and the Activities Specific Balance Confidence Scale [38]. Muscle tone will be measured using the Modified Ashworth Scale [39]. Lower limb strength and functional ability will be measured using the 5 times Sit to Stand test [40]. Quality of Life will be measured using the Stroke Specific Quality of Life Scale [41]. Details of all of these standardised outcome measures, their reliability and validity can be found in our case study paper [13]. The questionnaires will be self-administered by the participants prior to the assessment and all other clinical assessments will take approximately 30 minutes to complete.

### 2.6 Intervention

The intervention decribed below has been completed in line with the TIDieR checklist [42].

#### 2.6.1 Intervention title

Neuropilates remotely supervised online class for stroke survivors.

#### 2.6.2 Materials and procedures

Participants will be asked to participate in exercises remotely but in an online group setting with the other participants visible on screen and targeted at improving their posture, core stability, flexibility, abdominal and pelvic strength and balance. These exercises will be pitched at a beginner level focused on the core elements of neuropilates and progressing week on week as appropriate. The only materials required for the participants will be a chair, a theraband and a tablet electronic device, all of which will be provided at assessment. The instructor will require a laptop, wireless headphones with a microphone and a professional "Zoom" account. The neuropilates class will include a warm up, cool down and neuropilates exercises in line with the teaching of APPI ® (the Australian Physiotherapy and Pilates Institute). Examples of neuropilates exercise sets modified for the sitting and standing position are included in Table 2. In order to be adept at instructing neuropilates exercises such as these highlighted above, it is recommended that any interested Physiotherapist completes a qualification in pilates instruction with APPI and then completes the “neuropilates” CPD module, available here: https://appihealthgroup.com/buy/neuro-pilates/

**Table 2:** Examples of Neuropilates exercises: Adapted from APPI ® pilates exercises. Full instructions, technique, breathing, imagery and progressions of available from APPI ® courses for pilates instructors. All exercises shown with support of chair available as needed.

	Pelvic tilting and neutral spine alignment with transversus abdominis activation
	Postural setting with imagery for the whole body
	Weight shifting and stepping with weight transference
	Calf raises progressing to alternate calf raises
	“Standing Clam” in level 1 progressing to level 2 pictured here
	“Side leg lift” in standing level 1
	“One leg circle” in standing level 1
	“One leg stretch” in standing level 1 progressing to level 2 pictured here
	Stride stance with upper limb movements
	“Mermaid” in sitting
	“The offering” in sitting
	“Spine Twist” in sitting
	“Hundreds” modified to a sitting position with leg lifts as appropriate
	Sit to stand with focus on postural alignment and normal movement patterns progressing to no use of upper limbs
	Squats, wide squats, stepping into wide squats, sustained squats all with focus on neutral spine alignment and upper body postural cues

#### 2.6.3 Who will provide the intervention

A chartered Physiotherapist who holds a pilates qualification from APPI ® with 11 years' experience in stroke rehabilitation and 10 years’ experience in pilates instruction will conduct the online classes. The instructor has also undergone APPI ® modules in “neuropilates” and “3d standing pilates”.

#### 2.6.4 How and where will the intervention take place

The intervention group will attend a remotely supervised, live, online, neuropilates group exercise class from their homes via the online platform "Zoom". There will be an average of 4 participants per group.

#### 2.6.5 When and how much

The intervention will be delivered once a week over 6 weeks. Classes will be recorded and participants will be asked to complete the same class on their own twice more during the week. Classes will last 1 h. Initially, exercises will be pitched at a beginner level but exercises will get more challenging with higher repetitions, longer holds or less support in standing positions as the weeks progress. Previous studies investigating pilates in various clinical conditions have indicated that pilates interventions have proven effective if completed two to three times a week from periods of 4 weeks up to 12 weeks [[43], [44], [45]]. Once a week supervised with 2 more sessions unsupervised over 6 weeks was chosen as a timeframe that was deemed replicable in a busy rehabilitation “real-world” setting with budgetary, staffing and time constraints that may also prove effective in research settings, thus improving the replicability of the intervention.

#### 2.6.6 Tailoring and modifications

The Physiotherapist will use their clinical judgement throughout to tailor and modify the exercises to meet the needs of the individual participant. These modifications may include altering starting positions, recommended range of motion and level of support in standing to name a few.

#### 2.6.7 Adherence

Reminder texts will be sent out to each participant the morning of each class. After each class, the recording of the class will be emailed to each participant or their designated technological support person with a reminder to complete the recorded class twice during the week.

### 2.7 Control groups

There will be two control groups. Control group 1 will attend a live, remotely supervised, online, once weekly 60-min generalised exercise class from their homes over 6 weeks, facilitated via the online platform "Zoom" by the principal investigator. The exercises will be generalised with no specific focus on posture or alignment and may include stretches, simple strengthening exercises for upper and lower limbs and aerobic exercises such as seated and standing marching. These exercises are based on clinical experience as well as previous literature examining exercise classes in stroke patients [46]. Modifications will be made to the exercises as necessary including encouragement regarding appropriate range of motion or active assisted range of motion alternatives when needed. There will be an average of 4 participants per class. The participants will need a chair, a water bottle as a weight and a tablet device which they may receive on loan from the research team. The class will be instructed by a chartered Physiotherapist with 11 years’ experience in stroke rehabilitation.

Control Group 2 will be instructed in a tailored home exercise programme based on deficits found at assessment stage and will be asked to carry out these exercises at home, unsupervised, for 6 weeks. A paper copy of their exercises and if they possess a smart phone or laptop, a video copy of their exercises will be sent to them. They will be given a follow up phone call at week 3 to address any issues they may be having.

### 2.8 Statistics

We will analyse feasibility outcomes using descriptive statistics. Descriptive statistics will also be used to summarise the age, gender and functional independence of the participants in each group. Outcomes from clinical assessments will be completed using excel and paired T tests will be used to compare before and after data in each group. Anova tests will define if there are differences in the mean improvement or change in outcome scores between the three groups.

### 2.9 Data storage

All individual data will be identifiable during initial screenings, assessments and training sessions. Upon all assessment completion, data will be irrevocably anonymised. All data will also be coded to enable statistical analysis to take place. All data will be securely stored on the ATU Sligo campus, with access limited to the principal researcher and supervisor alone. Hard copy data will be stored in a secure locked filing cabinet and electronic data will be stored on a secure encrypted computer, in a password protected file.

### 2.10 Safety measures for remotely supervised exercising in intervention and control groups

At the initial assessment stage, those participants that need one will be given an electronic tablet device on loan for the duration of the classes. The principal investigator will give the participant a tutorial on the use of the tablet at this stage. A detailed information sheet will also be given to all participants in the intervention group and control group 1 with instructions about how to log into the class, class schedule, exercise information and information regarding monitoring their own symptoms prior to attending classes and seeking medical advice when appropriate. Participants will also be given advice regarding exercising safely at home and when to stop exercising if they experience any pain, dizziness or shortness of breath. Participants will be given a safe home exercise checklist to reinforce this verbal advice (Appendix 1) and a disclaimer (Appendix 2). These forms are based upon the recommended forms from the Irish guidance document for virtual pulmonary rehabilitation [47]. They must read these and return a written agreement to the terms and conditions before commencement of the classes. During the first class, the class instructor may individually cue the participants regarding their set up at home if it is suboptimal, or may phone the patient after the class to discuss modifications to the set up, e.g, holding onto a solid chair during standing or creating more space around them, if necessary.

Additionally, as recommended by the authors of the Irish guidelines for virtual pulmonary rehabilitation [47], if a patient scores less than 67% on the Activities Specific Balance Confidence Scale, they will be advised to have another person present in the house during the online exercise class. Patients exercising on their own will be asked to supply an emergency contact number which will be held on file by the principal researcher. In the case of an emergency event during the class the protocol outlined in Appendix 3 will be adhered to. Any adverse events that do occur will be reported in line with HSE open disclosure guidelines [48].

### 2.11 11 ethics

Ethical approval (#872) has been gained from both the Sligo University Hospital and ATU Sligo Ethics Committees.

### 2.12 Dissemination

Individual participant results will be posted to them. Results of the trial will be written up and published in a peer review journal and presented at relevant conferences during the following year.

## 3 Discussion

This randomised controlled feasibility study will be the first of its kind to investigate online neuropilates training in stroke survivors. The evidence for exercise therapy in stroke is plentiful with aerobic exercise [49,50] and “mixed training” (circuit training, resistance exercises and task orientated training) [9] being recommended in most clinical guidelines to improve fitness, balance and walking. Neuropilates has achieved some preliminary outcomes of balance and gait improvements in a small-scale systematic review [18] but further investigation is warranted before it could be recommended in this population as part of a clinical guideline.

Why study neuropilates however? And will it be any better than aerobic training or mixed training? Theoretically, pilates could potentially improve postural stability and balance in stroke survivors just through strengthening core and lower limb muscles. However, many “mixed training” studies in the Cochrane review by Saunders et al., 2020 [9] also included strengthening. What does neuropilates add that is different? Two key principles of pilates exercises which are not present in other forms of exercise are “concentration”, which involves increasing kinaesthetic awareness through specific focus on the exercises and “centreing”, a consistent and repetitive focus on neutral postures and alignment. These principles may help to minimise compensatory patterns of movement, accept equal weight through both lower limbs and improve verticality perception, theoretically.

Why study online exercise? The Covid-19 pandemic has taught us many lessons about the way in which we can deliver our services. Even before the pandemic, investigations into group-based telehealth had commenced to attempt to improve attendance rates [51,52]. Over the course of the pandemic, data emerged to suggest that virtual cardiac and pulmonary rehabilitation produce outcomes that are comparable to face-to-face group programmes [53,54]. However, group based online interventions are considerably under studied in stroke survivors. Online group exercise has the potential to improve access for a wider group of people, particularly in rural settings. Rural inhabitants have been shown to attend significantly fewer cardiac rehabilitation sessions than urban inhabitants [55]. If we can prove that online group exercise sessions are safe, feasible and effective in this population, it could be a useful alternative to offer certain groups who would prefer to exercise from home.

The proposed feasibility study should therefore begin to address the literature gaps highlighted above. It will provide Physiotherapists and instructors with a feasible, validated and scientifically evaluated pilates programme with suggested exercise modifications and safety considerations. It will also pave the way for larger randomised controlled trials that will be adequately powered to assess the effectiveness of the intervention. The results of the proposed study may also help to develop theories explaining the effects of pilates training in this population.

## Funding

This research has been undertaken as part of a funded PhD programme thanks to ATU Sligo's Masters Scholarship scheme. Sligo University Hospital Seed Grant will fund a number of electronic tablet devices for loaning to participants.

## Declaration of competing interest

The authors declare that they have no known competing financial interests or personal relationships that could have appeared to influence the work reported in this paper.

## Data Availability

No data was used for the research described in the article.

## APPENDIX I Safe Exercise Checklist

### Safe Exercise Checklist:

To keep yourself safe from harm or injury while exercising please ensure you have the following:

1) A stable surface to put your computer device on
2) Ample safe space to do the demonstrated exercises – enough to stretch both arms up and out fully and step in front and to the side without hitting anything
3) A family member, friend or carer present if advised
4) A supportive chair for exercising from and resting on
6) A glass of water or water bottle
5) No rugs, mats or electrical cords near you which may be a trip hazard
7) No pets present that may cause you to trip or fall.

If you experience any of the following symptoms, stop exercising immediately and inform me:

Chest pain.

Dizziness or feeling faint.

Extreme shortness of breath.

## APPENDIX II DISCLAIMER

Disclaimer:

I agree and consent to the following:

I am voluntarily participating in a 6-week Online Home-Based Exercise Programme as part of a clinical trial being conducted by ATU Sligo.

I understand that this is a novel intervention and the background and benefits of the programme have been explained to me.

I understand that when participating in any exercise there is a potential risk of injury.

I am taking part at my own risk and assume all risk of injury to myself.

ATU Sligo and the physiotherapist on this programme accept no liability.

I have read and will adhere to the Safe Home Exercise Checklist.

Signed:_________________________

Print Name:_____________________________

Date:___________________________________

## Appendix III Protocol in case of the occurrence of an adverse event

Protocol in case of the occurrence of an adverse event:

1. The class is stopped
2. All patients except for the unwell patient exit the platform.
3. The person in the house with the patient is advised on how to deal with the patient or the patient's emergency contact person is notified by the class instructor
4. The class instructor will have all eircodes of the participants readily available in case they need to contact an ambulance.
5. The class instructor will remain in contact with the patient via videolink until they are stable and/or have received medical attention
